# Comparative Study of the Solid-Liquid Interfacial Adsorption of Proteins in Their Native and Amyloid Forms

**DOI:** 10.3390/ijms232113219

**Published:** 2022-10-30

**Authors:** Ágnes Ábrahám, Flavio Massignan, Gergő Gyulai, Miklós Katona, Nóra Taricska, Éva Kiss

**Affiliations:** 1 Laboratory of Interfaces and Nanostructures, Institute of Chemistry, Eötvös Loránd University, Pázmány Péter Sétány 1/A, H-1117 Budapest, Hungary; 2 MTA-ELTE Lendület “Momentum” Peptide-Based Vaccines Research Group, Eötvös Loránd University, Pázmány Péter Sétány 1/A, H-1117 Budapest, Hungary; 3 ELKH-ELTE Protein Modelling Research Group, Institute of Chemistry, Eötvös Loránd University, Pázmány Péter Sétány 1/A, H-1117 Budapest, Hungary

**Keywords:** protein adsorption, amyloids, QCM technique, wettability, AFM imaging

## Abstract

The adhesive properties of amyloid fibers are thought to play a crucial role in various negative and positive aggregation processes, the study of which might help in their understanding and control. Amyloids have been prepared from two proteins, lysozyme and β-lactoglobulin, as well as an Exendin-4 derivative miniprotein (E5). Thermal treatment was applied to form amyloids and their structure was verified by thioflavin T (ThT), 8-Anilino-1-naphthalenesulfonic acid (ANS) dye tests and electronic circular dichroism spectroscopy (ECD). Adsorption properties of the native and amyloid forms of the three proteins were investigated and compared using the mass-sensitive quartz crystal microbalance (QCM) technique. Due to the possible electrostatic and hydrophobic interactions, similar adsorbed amounts were found for the native or amyloid forms, while the structures of the adsorbed layers differed significantly. Native proteins formed smooth and dense adsorption layers. On the contrary, a viscoelastic, highly loose layer was formed in the presence of the amyloid forms, shown by increased motional resistance values determined by the QCM technique and also indicated by atomic force microscopy (AFM) and wettability measurements. The elongated structure and increased hydrophobicity of amyloids might contribute to this kind of aggregation.

## 1. Introduction

Amyloid fibers, referred to as fibrillar proteins or peptide aggregates with a highly organized cross-β structure, were originally regarded as misfolded products that lead to human disorders, including neurodegenerative diseases, diabetes, and amyloidosis. In these so-called amyloid-associated or deposition diseases, insoluble amyloid aggregates are deposited extra- or intracellularly in tissues and organs. In all cases, aggregation is attributed to the misfolding of a specific protein.

However, during the last decade, increasing evidence has suggested that amyloid fibers exert multiple positive functional roles in processes occurring in the human body and smaller organisms [1,2,3], such as information transport and signaling or biofilm formation [4,5]. They are involved in the storage of human peptide hormones in amyloid granule form [6], the surface adhesion modulation in case of bacteria and algae (Taglialegna et al., 2016) [7] or forming chemically robust adhesive structures in fungi that are suggested for surface functionalization in biosensing applications [8]. Investigation of various functional amyloids, especially into how toxicity is avoided, may provide insights into the prevention of amyloid toxicity in amyloidosis [9].

These examples—among others—draw attention to the importance of understanding the adhesive properties of amyloids. Adsorption of a dissolved component at solid/liquid interface can be qualitatively/quantitatively characterized by several complementary analytical techniques, such as atomic force microscopy (AFM) [10], total internal reflection fluorescence microscope (TIRF) [11,12], in situ label-free optical techniques [13,14,15], polarization modulation–infrared reflection–adsorption spectroscopy (PM-IRRAS) [16] and quartz crystal microbalance (QCM) [17,18].

The QCM method, due to its high mass sensitivity and the possibility of in situ observation of the process, is widely used [19,20] in the field of proteins. It can be applied effectively for real-time monitoring of changes in thin films and measurements of adsorption and desorption of molecular layers, as well as for measurements of self-assembling monolayers. The method can be used to monitor the swelling and cross-linking of polymers in the liquid phase [21,22,23,24] or evaluate cell membrane-polymer interactions [25,26]. QCM has also been utilized in the study of protein amyloid formation. The investigation of fibril formation of Ure2p and glucagon [27,28] or degradation, as well as inhibition of amylin aggregates, could be followed with the aid of functionalized sensor surfaces [29,30]. The amyloid β protein, prone to forming neurotoxic assemblies, is also a subject of numerous studies using QCM. The detection of its oligomers is an important step in the early diagnostics of Alzheimer’s disease [31,32].

The focus of the present work is the comparative study of the adhesive behavior of native and amyloid forms of proteins. Globular proteins, lysozyme, β-lactoglobulin and an Exendin-4 derivative miniprotein (E5) were used as models representing various molecular sizes and compositions but having the common tendency to form amyloids. The effects of concentration and pH on the adsorption of the proteins onto QCM crystal surfaces were studied. The optimum conditions for the preparation of amyloid fibrillar structures were defined with the aid of fluorescence spectroscopy, circular dichroism spectroscopy and atomic force microscopy. To shed some light on the structural changes happening in the adsorption layer, the mass change was extracted from the contribution of the viscoelasticity of the layer in the interpretation of QCM results. The adsorption studies were completed by morphological characterization of the adsorbed layers by wettability and AFM studies.

## 2. Results and Discussion

### 2.1. Characterization of the Amyloid Formation

#### 2.1.1. Electronic Circular Dichroism Spectroscopy (ECD) Studies of the Protein Structure

**Figure 1:**
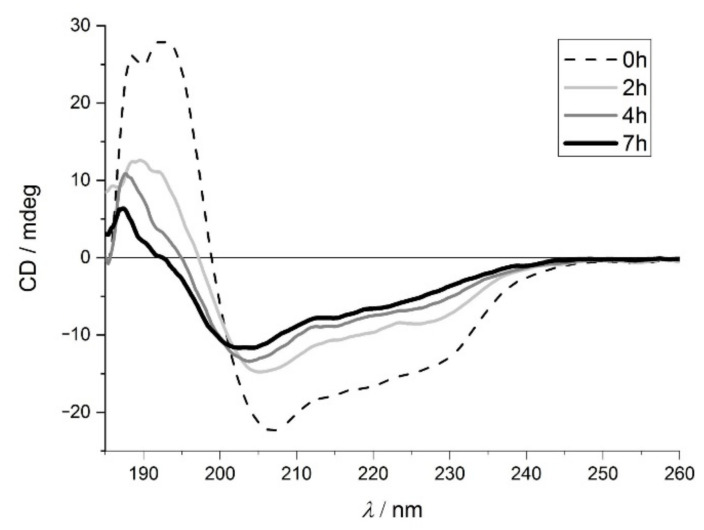
Far-UV electronic circular dichroism spectroscopy (ECD) spectra of lysozyme after 0 h (dashed line), 2 h (light grey), 4 h (grey) and 7 h (black) incubation.

Conformational changes of lysozyme were examined by ECD spectroscopy in the far-UV region. During the thermal treatment, a significant change can be detected in the globular protein’s structure (Figure 1). The native protein has a positive band at around 191 nm, a negative band at 207 nm and a wide negative band between 218–230 nm, which is characteristic of the well-folded protein with high α-helical content. This was confirmed by the BeStSel method [33] suitable for determining the individual components of the secondary structure; the native structure was 59% α-helix, see Table S1. The spectra indicate a significant change in the structure after 2 h incubation. With further heat treatment up to 7 h, the positive and negative peaks shifted to 187 nm and 204 nm, respectively, and mixed structures were observed (Table S1), which is consistent with previous literature results [34]. 

**Figure 2:**
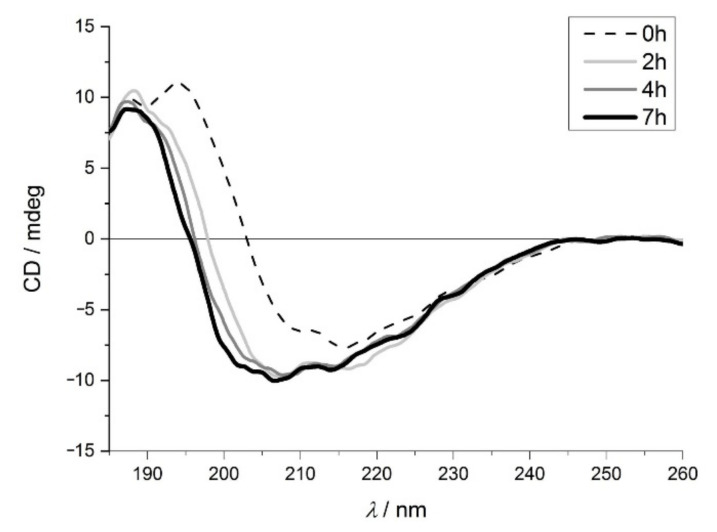
Far-UV ECD spectra of β-lactoglobulin after 0 h (dashed line), 2 h (light grey), 4 h (grey) and 7 h (black) of incubation.

The structural change during amyloid formation for β-lactoglobulin is shown in Figure 2. In the initial, native structure of β-lactoglobulin, the proportion of β-sheet secondary structural element is high, (about 20%-shown in Table S1), represented by the positive band at around 194 nm and negative band at around 216 nm. Nevertheless, a change in the shape of the spectrum can be observed during thermal treatment. The positive and negative peaks in the ECD spectrum shifted, which is consistent with previous literature results [35]. After 2 h the change is significant, while a gradual further shift is observed for samples after 4 and 7 h of incubation. Although the quantitative analysis of the ECD spectra (Table S1) does not clearly prove the occurrence of amyloid formation due to the simultaneous presence of mixed structures.

**Figure 3:**
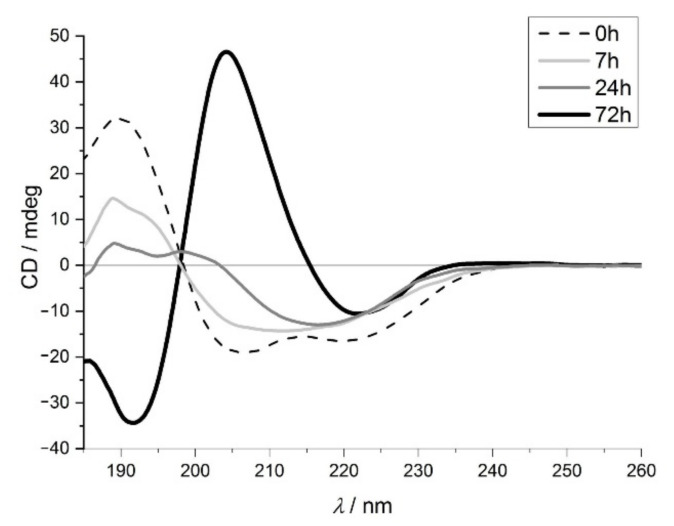
Far-UV ECD spectra of the Exendin-4 derivative miniprotein (E5) after 0 h (dashed line), 7 h (light grey), 24 h (grey), 72 h (black) incubation.

The Exendin-4 derivativeminiprotein (E5) [36] is an ideal model to study the amyloid formation with ECD spectroscopy because native and amyloid forms have very different spectra [37]. Due to its small size (only 25 amino acids) and the low number of secondary structural elements, an ECD spectrum characteristic of an unmixed structure can be detected, which was confirmed by the quantitative analysis (Table S1). In the case of the native form the spectrum has the classic features characteristic of an α-helix (positive-negative band pair at ~190 nm and ~205 nm and a negative band at 222 nm), because of the α-helix content is high (70%) while in the amyloid state the positive-negative band pair appears at 205 nm and 220 nm. After 72 h of incubation time (Figure 3) the amyloid formation was complete (the β-sheet content is high, about 86%-see Table S1)).

#### 2.1.2. Thioflavin T (ThT) Fluorescence Test

The ThT test is one of the most frequently applied methods to detect amyloid structures, since their fluorescent properties are altered in the presence of such fibrils. As the dye binds to the β-sheets of the amyloid, the fluorescence emission intensity is enhanced, while the wavelength of the emission peak suffers a considerable redshift (482 nm compared to free ThT’s 427 nm). In an amyloid-containing medium, excitation takes place at 450 nm, at which point the excitation peak may play a role in lowering the emission of the free dye via the quenching effect [38,39]. The suspected reason for these changes is the immobilization of benzylamine and benzathiol rings within the molecule. In solution, the two rings can rotate freely around the carbon-carbon bond connecting them, resulting in quenching of excited states. Contrarily, when bound to amyloid fibrils this rotation is blocked, resulting in maintained excitation, thus enhancing emission [40,41].

**Figure 4:**
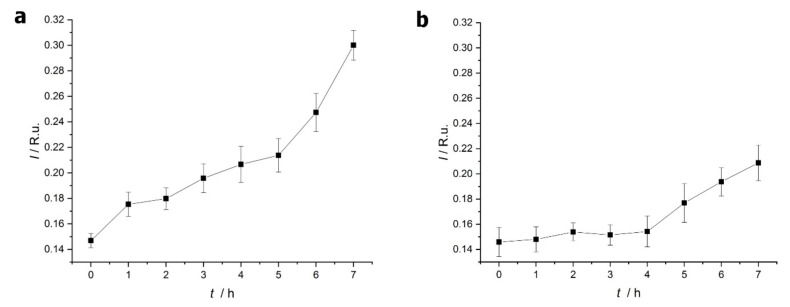
Fluorescence intensity (in water Raman units) of thioflavin T (ThT) with lysozyme (**a**) and β-lactoglobulin (**b**) samples as a function of thermal treatment time. The error bars represent the 95% confidence intervals. (Line is just to lead the eye).

During the amyloid formation, ThT was applied as an indicator of the protein structural change by measuring the sample fluorescent intensity. The fluorescence peak area of lysozyme and β-lactoglobulin are shown in Figure 4a,b as a function of incubation time. The fluorescence intensity increases with time for both proteins, but the increase is more pronounced for lysozyme. These observations suggest structural changes and fibril formation during the reaction. However, in the case of lysozyme, the fluorescence intensity change is monotone, while two ranges are seen for β-lactoglobulin. In the latter case, the intensity starts increasing after 3 h of thermal treatment.

Contrary to lysozyme and β-lactoglobulin the interaction of ThT with the amyloid of E5 miniprotein did not lead to increased fluorescence. This ThT silent behavior of E5 has been previously reported [37].

#### 2.1.3. 8-Anilino-1-Naphthalenesulfonic Acid (ANS) Fluorescence Test

ANS is a small-molecule extrinsic fluorophore that emits a strong fluorescence signal when located in a hydrophobic environment, accompanied by a blue shift from 515 nm to 480 nm [42]. It is extensively used to demonstrate the protein-folding intermediates, mainly amyloid fibers [43], and to identify hydrophobic patches on the protein surface.

**Figure 5:**
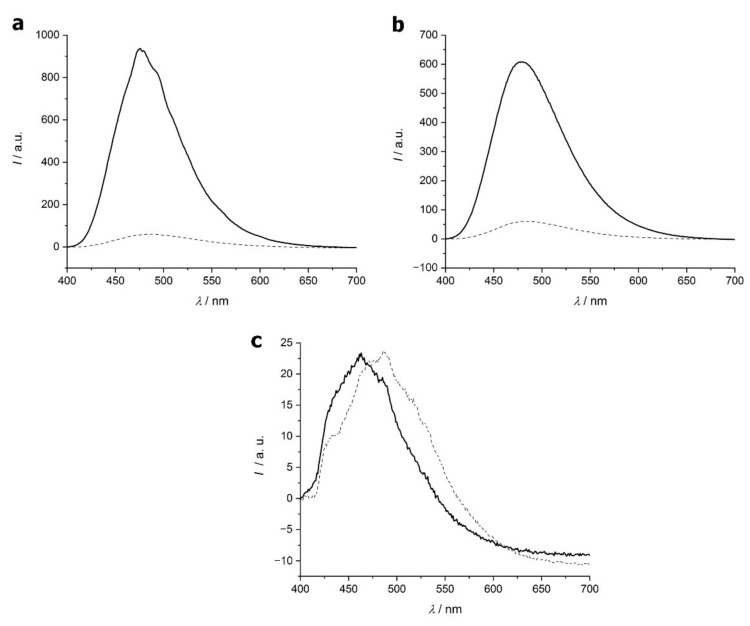
Fluorescence intensity of 8-Anilino-1-naphthalenesulfonic acid (ANS) in the presence of lysozyme (**a**), β-lactoglobulin (**b**) and E5 (**c**). Native proteins are indicated with dashed line and amyloid forms with a solid line. The concentration of ANS was 5 mM, lysozyme and β-lactoglobulin were 1.0 g/L and E5 was 0.59 g/L.

Surface hydrophobicity of water-soluble proteins at 25 °C is generally low because, in folded globular proteins, hydrophobic residues are located inside the tightly folded structure. The heat treatment leading to the formation of amyloid fibers results in a significant change in the folding, and therefore the surface polarity. The influence of this structural change on the ANS binding is detected as changes in fluorescence intensity in Figure 5a,b for lysozyme and β-lactoglobulin. The fluorescence intensity of both proteins following the 7 h thermal treatment is significantly increased compared to that of their native form, supporting amyloid formation. At three ANS concentrations, the fluorescence intensity was also studied as a function of protein concentration (Figure S1). A notable increase in fluorescence intensity for both proteins can be observed when higher amounts of ANS are applied, and also increases with protein concentration.

The emission of ANS in the presence of E5, however, did not show intensification following heat treatment induced amyloid formation (Figure 5c). It can be observed however that the peak for the native E5 is at 486 nm, while the amyloid’s is at 463 nm. That blue shift of the maximum emission of ANS is a sign that the dye binds to the hydrophobic protein [44].

Despite the lack of increased fluorescent intensity, this blue shift hints at some conformational change occurring in accordance with the increased amount of β-sheet detected by ECD. A possible explanation can be that the given size of hydrophobic patches on the amyloid fiber surface required for significant enhancement of ANS fluorescence [44] is not available due to the small number (4) of hydrophobic amino acids in E5 [45,46].

#### 2.1.4. Atomic Force Microscopy (AFM) of Amyloid Fibers

The structural elements of protein solutions in the nm range were investigated visually by AFM. Protein samples were deposited onto freshly cleaved mica surfaces and measured in dry form in air.

**Figure 6:**
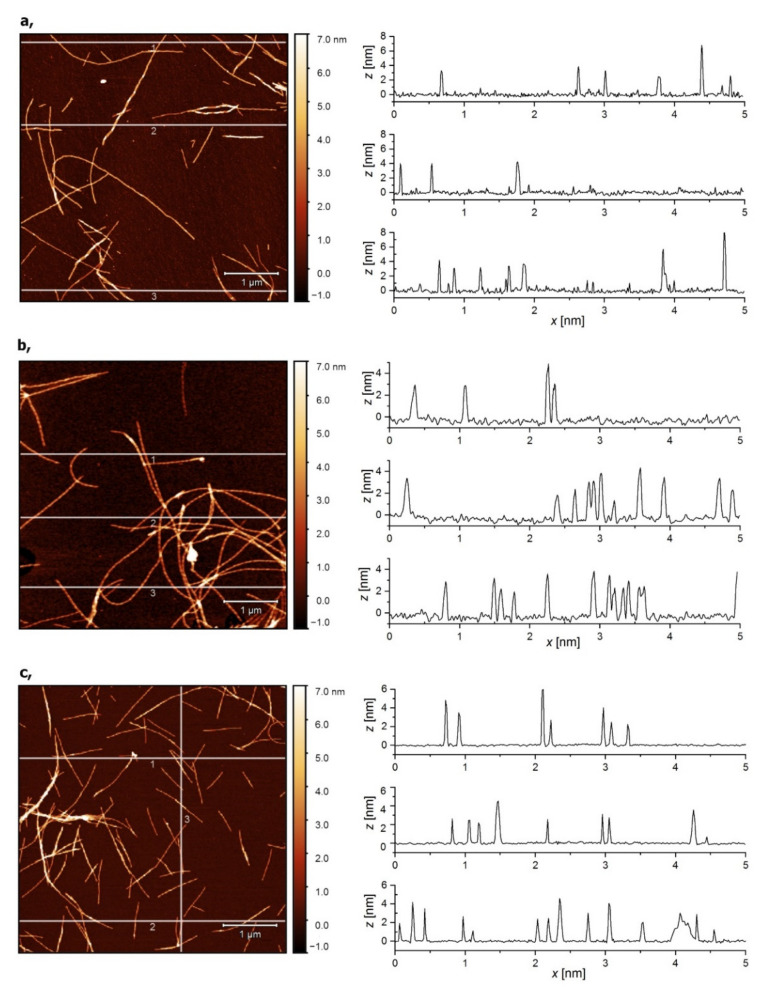
Atomic force microscopy (AFM) images of protein amyloid fibers obtained after heat treatment of lysozyme (**a**) and β-lactoglobulin (**b**) for 7 h and E5 (**c**) solution for 72 h with cross-section profiles at three selected positions (1, 2, 3) in each image.

No structural objects could be detected in the images obtained from initial protein solutions of lysozyme, β-lactoglobulin and E5. Similarly, no, or very few, particles or filaments were observed after the first hours of heat treatment of protein solutions. The 7 h heat treatment of lysozyme and β-lactoglobulin solutions, however, resulted in well-developed fibers as the AFM images showed. A comparably large number of fibers were found in the E5 solution after 72 h of incubation. The amyloid formation of the three proteins is illustrated in Figure 6. Cross-section profiles at three selected positions of each image are also displayed to allow the determination of the thickness of the amyloid fibers.

Typically, long fibers (micrometer range) were observed with a diameter in the range of 3–5 nm. The detailed results of the image analysis are summarized in Table S2.

Despite the inconclusive results of the ThT and ANS tests, it was found that the E5 miniprotein also forms long, well-developed fibers following incubation under given pH and temperature conditions. The β-rich structure of these amyloids is clearly proven by ECD. Based on the above results, 7 h of incubation for lysozyme and β-lactoglobulin and 72 h for E5 were selected for the preparation of amyloids used in the further experiments.

#### 2.1.5. Zeta Potential

Electrostatic forces may play an important role in the protein adsorption on the gold surface. Therefore, zeta potential measurements were performed to gain information about the protein charges. Zeta potential values summarized in Table 1 of the lysozyme and β-lactoglobulin change according to the expectation with the different pH conditions. Lysozyme is positively charged below its isoelectric point (i.e.p., pH = 11.1) and more highly charged at pH 2. The i.e.p. of β-lactoglobulin is 5.4; hence, the zeta-potential is positive at pH 2, but negative at pH 7. The zeta-potential values are similar to those obtained previously for lysozyme [47] and β-lactoglobulin [48] as well.

The charging character of amyloids shows the same tendency as the native proteins. Although the values are higher in some cases, the sign corresponds to the positive or negative character of the native proteins observed under acidic and neutral conditions. The highly increased positive zeta potential of lysozyme amyloid at pH 2 is the same as that reported recently (48.7 mV) [49,50]. The zeta potential of lysozyme amyloid is decreased, but still positive at pH 7.

β-lactoglobulin amyloid is characterized by a high positive zeta potential at pH 2, which changes to a significant negative value at pH 7. Similar behavior was found for β-lactoglobulin fibers by Peng et al. [51].

The zeta potential of E5 in its native form was obtained as (16.2 ± 0.9) mV, while that of the amyloid form is (10.5 ± 0.1) mV, determined at pH 4.1, which was used for further investigations.

### 2.2. Protein Adsorption on Gold Surface

#### 2.2.1. Quartz Crystal Microbalance (QCM) Studies of Adsorption

**Figure 7:**
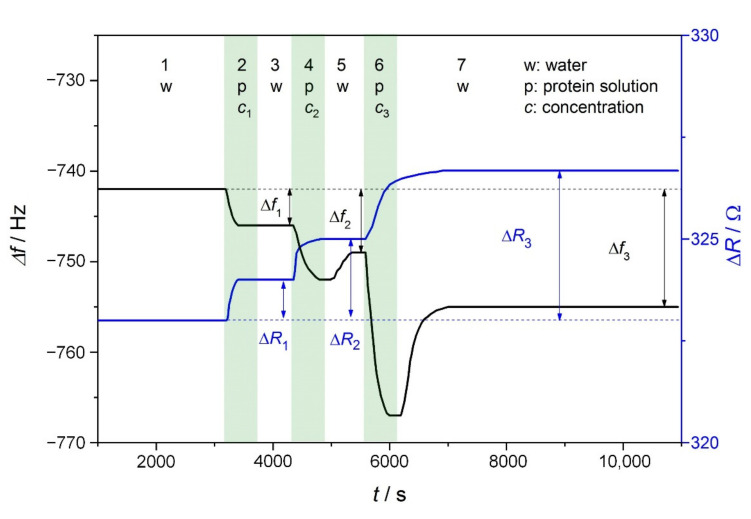
Changes in the resonance frequency (Δ*f*, black line) and resonance resistance (Δ*R*, blue line) of the quartz crystal microbalance (QCM) crystal over time (*t*). Both parameters are 0 for a pure crystal in contact with air. In step 1, the baseline is obtained with water. In steps 2, 4 and 6, protein solutions of increasing concentration are in contact with the crystal (*c*_2_ = 10*c*_1_, *c*_3_ = 100*c*_1_). In steps 3, 5 and 7, the crystal surface is rinsed with water.

Adsorption of proteins and amyloids onto the gold surface was investigated using the QCM technique. The concentrations of protein solutions were increased step by step from 0.001 to 1 g/L in 10-fold increments. The frequency and resistance changes were simultaneously detected; a typical curve is demonstrated in Figure 7. Each adsorption process was continued until a steady state was reached and was followed by a rinsing period as long as the frequency was stabilized.

Frequency change (Δ*f*) and change of motional resistance (Δ*R*) of the QCM crystal with the adsorbed protein layer are compared for two pH values of the adsorption. Applying different pH conditions is a way to modify the polarity of the gold surface of the QCM crystal and the charge character of the protein and allow the estimation of the contribution of electrostatic interaction to protein/amyloid binding. Jachimska et al. have investigated the surface polarity (zeta potential) of the gold QCM sensor as a function of pH [48]. Their study showed that the i.e.p. of the gold surface is 3.4. We selected pH 2 and 7 to study the protein adsorption where gold is cationic or anionic, respectively. Lysozyme is cationic at both pH values (i.e.p., pH = 11.1), while β-lactoglobulin and E5 with i.e.p.s in the mild acidic range (i.e.p., pH = 5.4 and 4.8, respectively) change their charge character by varying the pH from 2 to 7. QCM measurements with E5 were performed at pH 4.1 due to the optimum amyloid formation at that pH.

The frequency and resistance changes measured during the adsorption of proteins and their amyloids are summarized in Table 2, Table 3 and Table 4 for lysozyme, β-lactoglobulin and E5, respectively.

The decrease in frequency is proportional to the increased mass of the crystal due to protein adsorption. It is important to note that Δ*f* values characterize the tightly adsorbed mass in our case, since the values were determined after definite rinsing periods where the viscosity and density contributions of the medium are the same before and after the deposition. The Sauerbrey model describes the change in resonance frequency as a result of added mass uniformly distributed on the surface forming an elastic (rigid) layer. The motional resistance, *R*, detected simultaneously, is a clear indicator of whether this condition is fulfilled or not. It is closely related to the energy dissipation of the oscillating crystal surface and provides insights into a film’s viscoelastic properties. High *R* values are recorded when a “soft” film is formed. The viscoelastic contribution is estimated to be important once Δ*R* > 3 Ω, in accordance with previous experiences where the ratio of motional resistance to frequency change is given as a limiting value of 0.24 Ω/Hz [52]. Considering the values collected in Table 2, Table 3 and Table 4, the formation of a rigid film can be supposed in most cases and hence, the adsorbed amount of protein could be obtained using the Sauerbrey equation. These adsorbed amount data are presented in Table S3. However, in some cases, high *R* values (in bold) were observed, indicating considerable viscoelasticity of the surface layer, preventing the determination of the adsorbed mass in the previous way. This property is mainly characteristic of β-lactoglobulin at high concentrations.

The frequency values show that there is definite adsorption of native lysozyme onto the gold surface, which increases with the protein concentration (Table 2). The adsorbed amount obtained is substantially higher at pH 7 compared to the case of pH 2 where adsorption could only be detected at concentrations of 0.1 g/L and above. This relation can be explained by electrostatic interactions which are favorable at pH 7 where the charge character of lysozyme and the solid surface are opposite.

The adsorption of lysozyme amyloid presents a similar behavior. Adsorption increases with concentration and higher values are determined under neutral conditions than in the acidic medium. This is in agreement with the zeta potential of amyloids (Table 1) presenting higher values but with the same sign as the native protein. This supports the importance of electrostatic interactions in the adsorption of lysozyme and its amyloid.

The frequency changes show noticeable adsorption of β-lactoglobulin onto the gold surface (Table 3). The adsorbed amounts are detectable even at the lowest concentration and further increase with the protein concentration in the medium for both native and amyloid forms. At pH 7, adsorption is promoted more than in acidic conditions reflected by the frequency values. Considering the electrostatic interaction derived from the zeta potential of the gold surface and the protein, this seems to be surprising. β-lactoglobulin is cationic at pH 2 and anionic at pH 7; therefore, its zeta potential has the same sign as the gold surface at both pHs. Given these facts, the main driving force of adsorption might not be the electrostatic interaction, since the measured values of the adsorbed quantities are in the opposite relation to what could be assumed based on charge relations. On the other hand, to evaluate the possible electrostatic interactions operating in protein adsorption, it has to be taken into account that zeta potential is just an overall parameter that does not reveal the charge distribution on the molecular surface. Therefore, electrostatic attraction cannot be excluded between the sensor surface and some parts of the protein leading to significant adsorption supposing the appropriate orientation of the protein.

In addition to that, hydrophobic interaction can also participate in the bonding, especially in the case of β-lactoglobulin, which is rich in hydrophobic amino acid sequences [53], and that structural feature results in possibly extended hydrophobic surface domains in the amyloid form as the increased ANS binding showed.

The E5 miniprotein also showed a significant change in the measured frequency during adsorption. The zeta potential of E5 in its native form is (16.2 ± 0.9) mV and that of the amyloid form is (10.5 ± 0.1) mV; therefore, it can be stated that the peptide is charged oppositely to the crystal surface at the applied pH 4.1 in both states. There is a definite affinity of E5 to the gold surface as the adsorbed amounts are in the same range as was obtained for β-lactoglobulin or lysozyme in spite of its much smaller molecular weight. Referring to its chemical composition, E5 is a rather hydrophilic protein. This means that electrostatic interactions play a dominant role in its adsorption onto the surface of gold.

**Figure 8:**
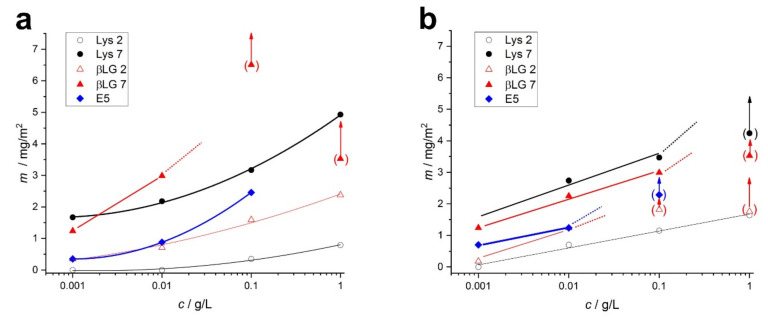
Adsorbed amount, *m* (mg/m^2^), of each protein (**a**) and its amyloid form (**b**) on QCM crystal surface measured at pH 2 (unfilled symbol) and 7 (filled symbol) at various concentrations. The arrows with the bracketed data points indicate where the determined adsorbed amount is underestimated.

The calculated surface concentrations of the adsorbed protein and the amyloids are displayed in Figure 8, allowing the direct comparison of the various proteins at different pHs.

The adsorbed amounts, calculated using the Sauerbrey model, that are influenced by the viscoelasticity of the layer (in brackets) are distinguished from the rest of the data. In these cases, high motional resistances were detected; therefore, the calculated surface concentrations are not realistic. According to a detailed analysis of soft films in liquids, the apparent mass as derived with the Sauerbrey equation is smaller than the film’s actual mass [54,55]. Voinova et al. call this the “missing-mass effect” [56]. These apparent masses are also displayed in Figure 8, with the arrows referring to the fact that those mass values are underestimated. As can be seen, the formation of soft layers mainly happens in the case of amyloid adsorption at higher concentrations (Figure 8b). In the case of native proteins, the adsorption layer with considerable viscoelasticity is obtained only from β-lactoglobulin under neutral conditions at a high concentration, possibly due to its aggregation.

#### 2.2.2. Wettability of the Adsorbed Surfaces

**Figure 9:**
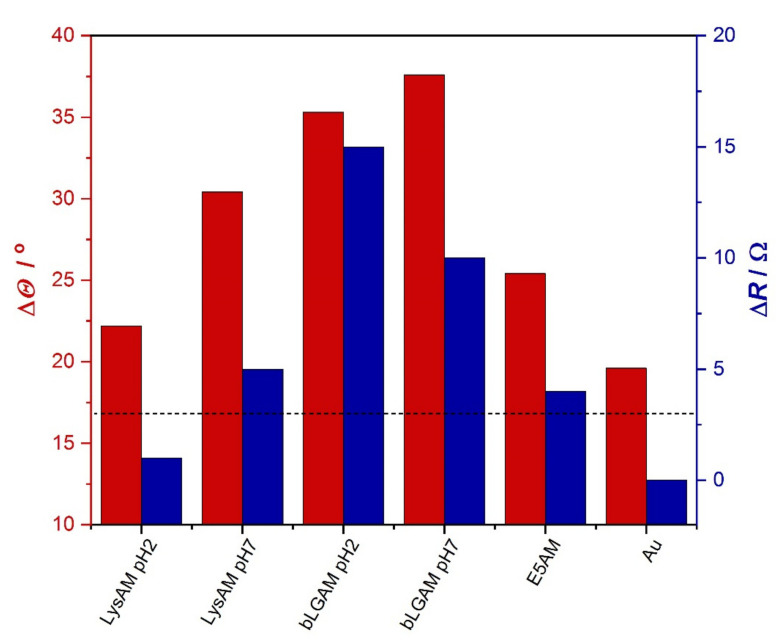
Water contact angle hysteresis (Δ*Θ*, red) on QCM crystal (Au) and with adsorbed protein layers on it as well as the motional resistance (Δ*R*, blue) of the corresponding crystal in QCM measurement. Δ*R* = 3 Ω value is indicated in dashed line.

Wettability measurements were performed on the QCM crystal surface following the native and amyloid protein adsorption. Advancing and receding contact angles of water were determined. The contact angles are in the range of 50–80 degrees, including the bare gold surface of the QCM crystal. The hysteresis (Δ*Θ*), which is the difference between the advancing and receding angles, represents the surface heterogeneity. Δ*Θ* of 20 degrees measured on gold surface is reasonable; since the crystal surface is not smooth, gold surfaces prepared by physical vapor deposition (PVD) have a roughness of about 1 nm [54,55]. Even higher hysteresis values were observed for crystal surfaces carrying the adsorbed amyloid layers, which refer to the irregular rough structure of the layer. Contact angle hysteresis and motional resistance values (Δ*R*) determined during the QCM measurements are displayed together in Figure 9. It is interesting to see that these values show a significant correlation. That means that systems with highly viscoelastic protein films, where the adsorbed amyloid fibers probably form a loose network-like structure, correspond to the surfaces presenting high roughness.

#### 2.2.3. AFM Investigation of the Adsorbed Surfaces

**Figure 10:**
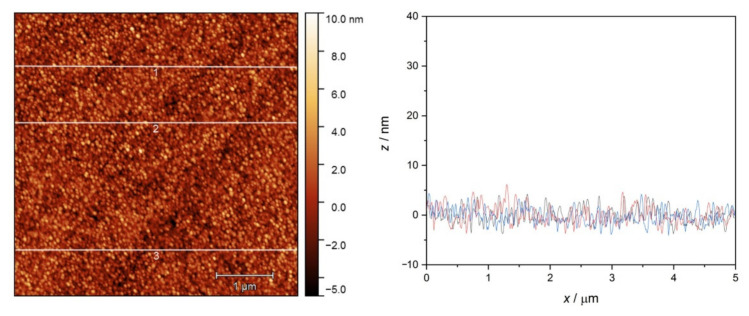
AFM image of the unmodified gold QCM sensor surface and line profiles at three selected positions (1 (black), 2 (red), 3 (blue)).

AFM images were obtained from the QCM crystal surfaces after the adsorption of native proteins or their amyloid. The surface of the pure gold-coated crystal is displayed with some characteristic cross-section profiles as a reference in Figure 10. The vapor-deposited gold coating proved to be a rough surface, with a roughness in the range of 3–5 nm.

**Figure 11:**
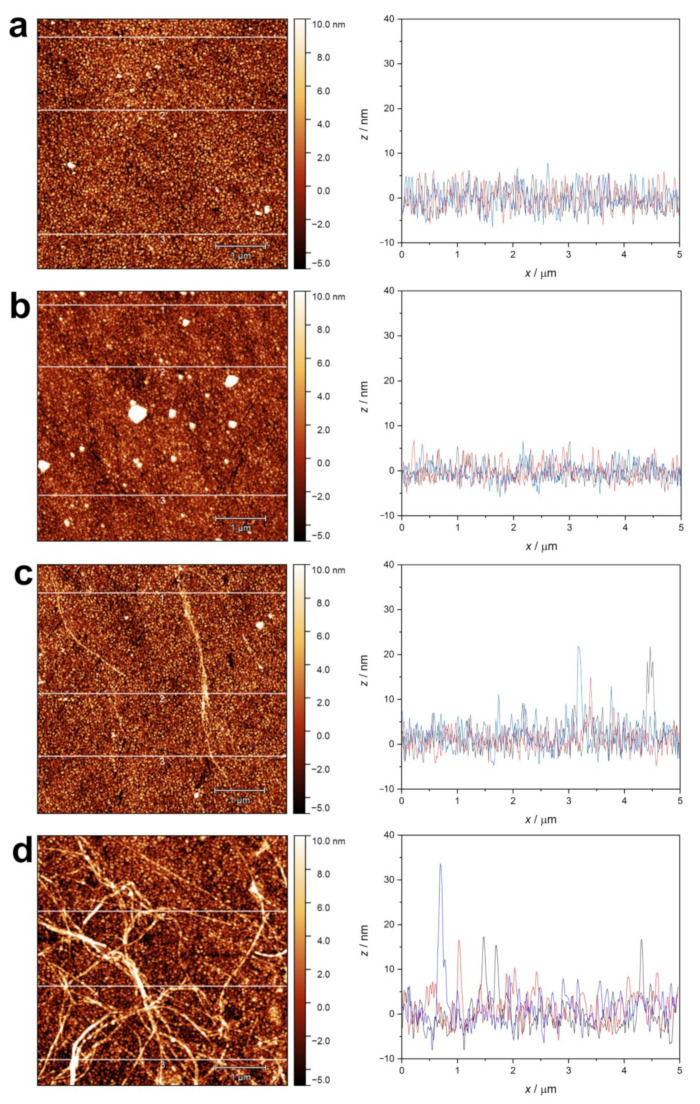
AFM topographic images of QCM sensor crystal surfaces after protein adsorption measurement. (**a**) native lysozyme at pH 2.0; (**b**) native lysozyme at pH 7.0; (**c**) lysozyme amyloid at pH 2.0; (**d**) lysozyme amyloid at pH 7.0. Line profiles at three selected positions (1 (black), 2 (red), 3 (blue)) are presented.

**Figure 12:**
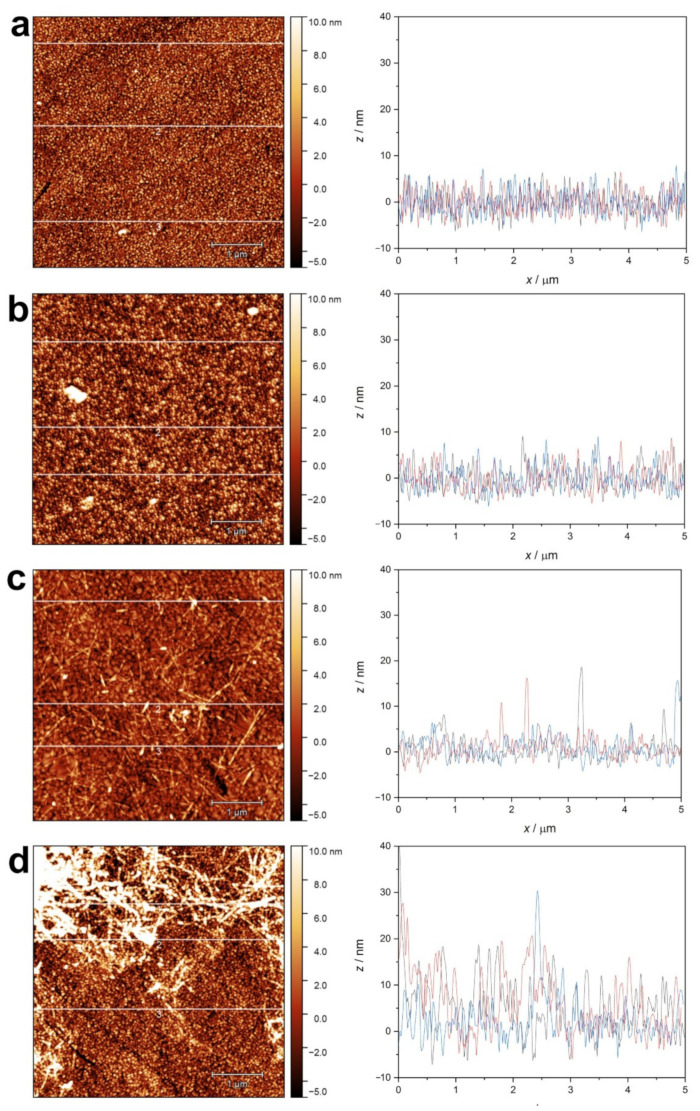
AFM topographic images of QCM sensor crystal surfaces after protein adsorption measurement. (**a**) native β-lactoglobulin at pH 2.0; (**b**) native β-lactoglobulin at pH 7.0; (**c**) β-lactoglobulin amyloid at pH 2.0; (**d**) β-lactoglobulin amyloid at pH 7.0. Line profiles at three selected positions (1 (black), 2 (red), 3 (blue)) are presented.

The adsorption layers of native lysozyme (Figure 11a) and β-lactoglobulin at pH 2 (Figure 12a) were found to be rather uniform, not differing significantly from the pure gold surface. Only the overall roughness increased to a small degree.

The surface morphology of the adsorbed layer in the case of native lysozyme (Figure 11b) and β-lactoglobulin at pH 7 (Figure 12b) also seems to be similarly uniform, except for the appearance of a few small aggregates.

In the case of lysozyme amyloid adsorption at pH 2 (Figure 11c), several individuals or bundled fibers could be observed. The higher amount of lysozyme amyloid adsorbed at pH 7 shows a tangled, network-like structure (Figure 11d). The cross-section profiles show structural elements in the vertical range of 20–30 nm.

The adsorption of β-lactoglobulin amyloid leads to increased roughness of the surface due to the adhesion of fibers (Figure 12c). Their surface density seems to be lower when adsorption occurs at pH 2, while a highly dense, crosslinked layer was formed at pH 7 (Figure 12d).

**Figure 13:**
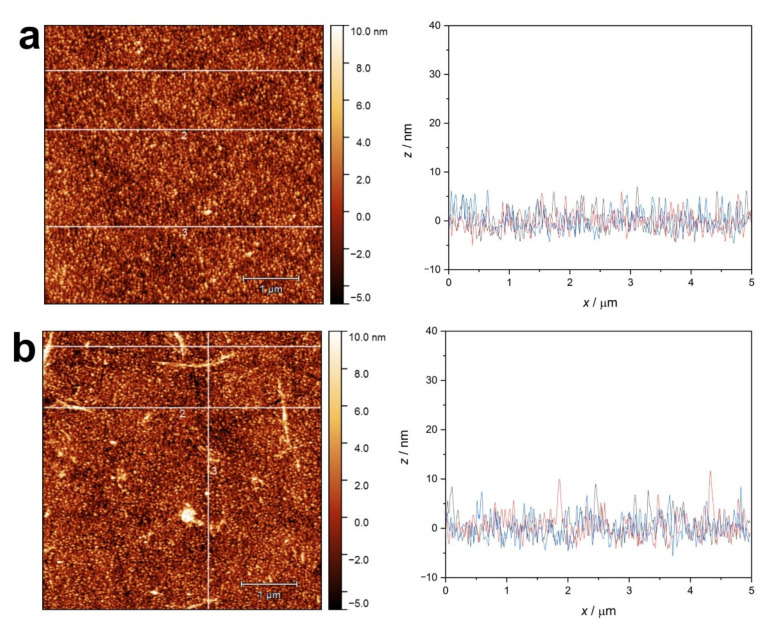
AFM topography images of QCM sensor crystal surfaces after adsorption of native E5 (**a**) and E5 amyloid (**b**). Line profiles at three selected positions (1 (black), 2 (red), 3 (blue)) are presented.

Similar but smaller differences were obtained in the case of E5 adsorption, comparing the native and amyloid covered surfaces (Figure 13). The surface morphology is quite even in the former case, but short fibers could be observed in the case of amyloid adsorption.

The above observation allows the conclusion that amyloid fibers holding notable surface hydrophobicity (supported by the ANS test) tend to adhere to the substrate and each other, as well as forming a thick layer with a tangled structure. These layers behave as viscoelastic films in the hydrated form as the high motional resistance indicates in QCM measurements. This behavior is more pronounced in the case where the adsorption is preferred, and the fibers are long.

## 3. Materials and Methods

### 3.1. Materials

Hen egg white lysozyme (>95%) was purchased from VWR International Kft. (Debrecen, Hungary), β-lactoglobulin AB (>90%) was obtained from Sigma-Aldrich Kft. (Budapest, Hungary). Exendin-4 (or Exenatide) derivative miniprotein (EEEAVRLYIQWLKEGGPSSGRPPPS) (a marketed drug for type-2 diabetes mellitus, E5) was produced by bacterial expression using the previously described protocol [57]. Specific parameters of the proteins were summarized in Table 5 and Table S1.

**Figure 14:**
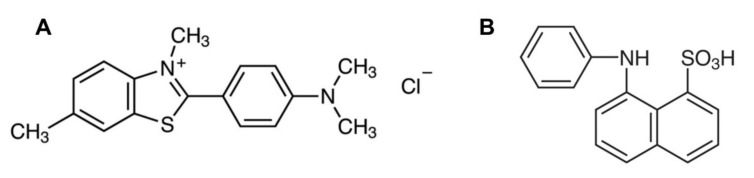
Chemical structure of ThT (**A**) and ANS (**B**) fluorescence dyes.

Thioflavin T (C_17_H_19_ClN_2_S, M = 316.86 g/mol, >98% purity; ThT) and 8-Anilino-1-naphthalenesulfonic acid (C_16_H_13_NO_3_S, M = 299.35 g/mol, >98% purity; ANS) dyes were obtained from Sigma-Aldrich Kft. (Budapest, Hungary). Their structures are shown in Figure 14.

The stock solution of ThT was prepared by dissolving 4 mg of solid ThT in 5 mL phosphate buffer. The solution was filtered through a 0.2 µm regenerated cellulose (RC) membrane, then its final concentration was determined spectrophotometrically (ThT has an absorption peak at 412 nm with a molar absorption coefficient of 36,000 $M^{-1}{\mathrm{cm}}^{-1}$) The filtered stock solution was freshly diluted to 50 µM with buffer before the fluorescence measurements.

The medium of the fluorescence measurements was 0.01 M phosphate buffer pH = 7 containing 0.01 M potassium dihydrogen phosphate, 0.01 M disodium hydrogen phosphate and 0.15 M sodium chloride solutions. All chemicals were purchased from Sigma Aldrich Kft., Budapest, Hungary and their purities were >98%.

Sodium phosphate buffer (pH = 7.4) was used to obtain the ANS working solutions with concentrations of 5 and 2.5 mM.

Double-distilled water checked by its electrical conductance (<5 mS) and surface tension (72.0 mN/m at (25.0 ± 0.5) °C) was applied for the experiments.

The pHs of the protein solutions were adjusted by hydrogen chloride (a.r., VWR International Kft., Debrecen, Hungary; HCl) and sodium hydroxide (a.r., VWR International Kft., Debrecen, Hungary; NaOH) solutions. Sodium chloride was also used during zeta potential measurements.

The QCM gold crystal sensors were washed with a 1:1:5 mixture of ammonia (25% aqueous solution, analytical grade, Molar Chemicals Kft., Halásztelek, Hungary), hydrogen peroxide (30% aqueous solution, analytical grade, Molar Chemicals Kft., Halásztelek, Hungary) and distilled water at 70 °C for 10 min, followed by treatment in an air plasma chamber (30 W, 0.2 mbar) for 10 min.

A 2% solution of the Deconex 20 NS (VWR International Kft., Debrecen, Hungary) was applied for cleaning the head of the QCM flow cell.

### 3.2. Preparation of Amyloids

Thermal treatment was applied to obtain amyloid structures from the given protein. Aqueous solutions of lysozyme (20 g/L) and β-lactoglobulin (2 g/L) with pH set to 2 were incubated at 90 °C for 1–48 h. Amyloids obtained after 7 h of incubation were selected for further experiments.

Amyloid of E5 was prepared by incubation of its aqueous solution with a concentration of 250 mM and pH set to 4.1 at 37 °C for 72 h.

### 3.3. Quartz Crystal Microbalance (QCM)

Protein and amyloid adsorption were studied using quartz crystal microbalance equipment (QCM200, Stanford Research Systems, Sunnyvale, CA, USA) on gold-covered sensor crystal surfaces. AT-cut quartz crystals were used, providing great mass sensitivity and a low temperature coefficient around room temperature.

The technique is based on the inverse piezoelectric effect. The fundamental frequency (*f*_0_) is determined by the crystal sheet’s *L_K_* thickness and the *v_tr_* transverse velocity of sound in the crystal (which is the function of its *ρ_q_* density and *µ_q_* elastic modulus):

(1) $$f_{0}=\frac{v_{tr}}{2\cdot L_{k}}\;$$

To interpret the operation of QCM, it is essential to assume that the transverse sound velocity *v_tr_* is the same in the deposited layer as in the quartz crystal. A change in the thickness of the quartz crystal implies a change in the frequency (Δ*f*) that can be measured with high accuracy. That change can also be clearly related to the change in mass (Δm) uniformly distributed on the active surface described by the Sauerbrey equation [59]:

(2) $$\Delta f_{m}=-\frac{2\cdot f_{0}^{2}}{\sqrt{\mu_{q}\cdot \rho_{q}}}\cdot \Delta m_{A}=-C_{f}\cdot \Delta m_{A}\;$$

where $C_{f}$ is the integral sensitivity or device constant, comprising *ρ_q_* density and *µ_q_* elastic modulus of quartz and *f*_0_ fundamental frequency of the crystal.

The vibration of a crystal also depends on the surrounding medium. The frequency of vibration decreases with increased density and viscosity of the liquid when the crystal is immersed in liquid. The function is described by the Kanazawa–Gordon equation [60]:

(3) $$\Delta f_{dv}=f_{0}{}^{\frac{3}{2}}\sqrt{\frac{\eta_{l}\cdot \rho_{l}}{\pi \cdot \mu_{q}\cdot \rho_{q}}}\;$$

where *η_l_* and *ρ_l_* are the viscosity and density of the liquid.

The device used for the measurements (SRS QCM200) detects and records resonance motional resistance (*R*) as well. These data are related to the adsorbed layer’s mechanical properties, such as the product of its density and viscosity, described by the Butterworth equation [61,62]:

(4) $$\Delta R=\frac{n\cdot \omega \cdot L}{\pi }\sqrt{\frac{2\cdot \omega \cdot \eta_{l}\cdot \rho_{l}}{\mu_{q}\cdot \rho_{q}}}\;$$

where *ω* is the angular frequency and *L* is the inductance for the unperturbed (dry) resonator.

The QCM sensor crystal was enclosed in a flow cell, connected to a syringe pump applying a liquid flow rate of 0.25 mL/min. Experiments were performed at (25.0 ± 0.1) °C. The crystal had a resonant frequency of 5 MHz, while the diameter, the sensitivity factor (*C_f_*) and the inductance of the electrode were 14 mm, 56.6 (Hz·cm^2^)/μg and 0.03 H, respectively [63]. During measurements, the resonance frequency (*f*) and the motional resistance (*R*) of the sensor crystal were recorded. For each system, measurements were performed at least in triplicate, and sample-to-sample reproducibility was within 15%.

### 3.4. Fluorimetry

The fluorescence of native and amyloid protein solutions was studied in the presence of ThT or ANS dyes. Fluorescence spectra were recorded using a VARIAN Cary Eclipse (Agilent Technologies Inc., Santa Clara, CA, USA) fluorescence spectrophotometer (right-angle geometry, 1 cm × 1 cm quartz cell). The temperature ((25.0 ± 0.1) °C) in the cuvette was controlled by a Peltier-type thermostat.

The examined sample was diluted to 1 g/L concentration with buffer and 30 µL of this solution was added to 3 mL 50 µM ThT solution, stirring intensively for a minute before the measurement. The diluted dye solution was freshly prepared because of its light sensitivity. The excitation wavelength was set at 440 nm (5 nm monochromator slit width). The emission was recorded between 450–600 nm (10 nm monochromator slit width) with an emission peak at 482 nm. Detector voltages were varied in the 600–800 V range. The spectra were normalized with Raman-scattering spectra integrals of pure water, recorded at the respective detector voltage (350 nm excitation, 360–420 nm emission)

The ANS dye test measurements were performed as described previously [42]. The concentrations of the protein solutions were in the range of 5–0.5 g/L. To begin the test, 15 μL of ANS solution was added to 3 mL of protein solution. Following the mixing, the sample was kept in the dark for 30 min at room temperature. An excitation wavelength of 380 nm was used for the samples with the wavelength of emission spectra in the 400–700 nm range. Monochromator slit widths were set at 5 nm for both excitation and emission.

The experiments were repeated twice, and the average was used in the calculation of the increase in emission.

### 3.5. Zeta Potential Measurement

The electrophoretic mobility of the samples was determined by Zetasizer Nano Z (Malvern Panalytical, Malvern, United Kingdom) apparatus at (25.0 ± 0.1) °C in 2 mM NaCl solution medium to ensure a constant ionic strength. Results were collected in triplicate. For calculating the zeta potential (ζ) from mobility values, Smoluchowski approximation was applied.

### 3.6. Electronic Circular Dichroism Spectroscopy (ECD)

Far-UV ECD measurements were carried out on a Jasco J810 spectrophotometer (JASCO Inc., Easton, PA, USA) in 1.0 mm quartz cuvette over the 185–260 nm wavelength range. In the case of E5 miniprotein, 10-fold (0.056 g/L) diluted samples were measured with ECD spectroscopy, while lysozyme and β-lactoglobulin samples were measured after diluting 8-fold (0.125 g/L)). Spectra were recorded with 50 nm/min scanning speed, 1 nm bandwidth and 0.2 nm step resolution, with three scans averaged for each spectrum. The temperature at the cuvette was controlled by a Peltier-type thermostat at (25.0 ± 0.1) °C. The ratio of secondary structural elements of the measured proteins in their native and amyloid state was determined from the measured ECD curves using the BeStSel program [33].

### 3.7. Wettability Measurements

The water contact angle of the deposited layers was determined by an optical contour analysis system (OCA15+, Dataphysics, Filderstadt, Germany). Measurements were performed in a closed, water vapor-saturated, temperature-controlled chamber at (25.0 ± 0.1) °C. The contact angle of a water droplet (5 µL) was determined on the untreated, cleaned gold QCM sensor surface and the dried protein amyloid-covered surfaces after QCM measurements. Maximum advancing (*θ*_A_) and minimum receding (*θ*_R_) angles were measured by increasing and decreasing the volume of the drop using a motor-driven Hamilton syringe [64]. The SCA20 software was utilized to analyze the drop contours of the recorded images and calculate the contact angle using the ellipse-fitting method. The measurements were carried out in triplicates.

### 3.8. Atomic Force Microscopy (AFM)

Protein amyloid structure formation after the thermal treatment was also confirmed by atomic force microscopy imaging. For this process, 50 µL of protein solution was deposited onto a freshly cleaved mica surface. After 10 min adsorption time, the substrate was gently rinsed in double distilled water and dried in vacuum before imaging in air at room temperature.

Following the QCM adsorption experiments, the dried gold sensor surfaces were also imaged with AFM.

Surface morphology was recorded with a FlexAFM microscope system (Nanosurf AG, Liestal, Switzerland), operating in dynamic mode. Tap150GD-G cantilevers (BudgetSensors Ltd., Sofia, Bulgaria) with a nominal tip radius of less than 10 nm were used for the measurements. Images were recorded over 5 μm × 5 μm window areas at 10 randomly selected locations with a resolution of 512 pixels/line. 

Representative line profiles have been extracted from the images to characterize the surface roughness of the samples.

## 4. Conclusions

Well-known proteins which can form amyloids under certain laboratory conditions were selected for the experimental study using the in situ mass-sensitive analytical technique, QCM. The comparison of adhesive properties of native proteins and their amyloids led to the finding that both forms result in a tightly bound adsorption layer on the gold surface. The results of QCM measurements are interpreted in the form of mass change, separated from the contribution of the surface viscosity of the layer.

The adsorption process was studied as a function of pH, which permits the evaluation of interactions involved in the binding and helps to shed some light on the surface properties of amyloid fibrils formed from different proteins.

Overall, it can be stated that the electrostatic driving force is important during adsorption; however, the zeta potential of the involved particles and that of the solid surface alone is not sufficient to describe it. This can be explained by the heterogeneous surface charge distribution of the proteins. This means that, depending on the orientation of the proteins, attractive interactions can occur even when the net charge would exclude them. Moreover, hydrophobic interactions can occur with apolar molecular segments turning towards the surface.

The latter is more pronounced in the case of amyloids that form a loose, network-like film with significant viscoelasticity and roughness in contrast with the rigid, even layer of the native proteins. This structural difference is more distinct with bigger proteins that form longer fibers, due to the preferred interactions between the surface and the exposed hydrophobic domains.

These observations, although made in a laboratory environment, can provide some contribution to the understanding of the formation of amyloid structures by aggregation.

## Figures and Tables

**Table 1:** Zeta-potential (*ζ*) of lysozyme and β-lactoglobulin as well as its amyloid forms determined at pH 2 and pH 7.

Protein	*ζ*/mV	
	pH = 2.0	pH = 7.0
Native lysozyme	29.1 ± 2.2	4.1 ± 0.7
Lysozyme amyloid	48.3 ± 2.2	26.4 ± 0.2
Native β-lactoglobulin	17.5 ± 5.2	−26.9 ± 1.3
β-lactoglobulin amyloid	52.5 ± 2.7	−26.8 ± 0.7

**Table 2:** Frequency (Δ*f*) and resistance changes (Δ*R*) determined by QCM measurement during adsorption of lysozyme at various concentrations and pHs.

pH	Lysozyme	*c*/g/L	Δ*f*/Hz	Δ*R*/Ω
2.0	NATIVE	0.001	0.00 ± 0.05	1.00 ± 0.10
		0.01	0.00 ± 0.07	1.00 ± 0.15
		0.1	−2.00 ± 0.32	1.00 ± 0.12
		1	−4.50 ± 0.50	0.50 ± 0.06
	AMYLOID	0.001	0.00 ± 0.06	0.00 ± 0.05
		0.01	−4.00 ± 0.42	0.50 ± 0.07
		0.1	−6.50 ± 0.71	1.00 ± 0.11
		1	−9.30 ± 1.10	1.00 ± 0.13
7.0	NATIVE	0.001	−9.50 ± 1.05	1.00 ± 0.08
		0.01	−12.33 ± 1.33	1.00 ± 0.12
		0.1	−18.00 ± 1.80	1.30 ± 0.15
		1	−28.00 ± 2.10	2.30 ± 0.20
	AMYLOID	0.001	−9.00 ± 0.97	0.00 ± 0.04
		0.01	−15.50 ± 1.25	1.00 ± 0.95
		0.1	−19.67 ± 1.74	1.67 ± 0.14
		1	−24.00 ± 2.05	5.00 ± 0.49

**Table 3:** Frequency change (Δ*f*) and resistance change (Δ*R*) determined by QCM measurement during adsorption of β-lactoglobulin at various concentrations and pHs.

pH	β-Lactoglobulin	*c*/g/L	Δ*f*/Hz	Δ*R*/Ω
2.0	NATIVE	0.001	−2.00 ± 0.25	1.00 ± 0.07
		0.01	−4.00 ± 0.53	1.00 ± 0.08
		0.1	−9.00 ± 0.95	2.00 ± 0.25
		1	−13.50 ± 1.21	2.00 ± 0.19
	AMYLOID	0.001	−1.00 ± 0.09	0.00 ± 0.04
		0.01	−7.00 ± 0.80	1.00 ± 0.09
		0.1	−10.33 ± 1.10	9.00 ± 0.99
		1	−10.00 ±1.02	15.00 ± 1.65
7.0	NATIVE	0.001	−7.00 ± 0.75	1.00 ± 0.10
		0.01	−17.00 ± 1.64	0.00 ± 0.07
		0.1	−37.00 ± 4.10	11.00 ± 1.52
		1	−20.00 ± 1.83	8.00 ± 1.26
	AMYLOID	0.001	−7.00 ± 0.78	1.00 ± 0.09
		0.01	−12.75 ± 1.30	1.00 ± 0.10
		0.1	−17.00 ± 1.60	2.30 ± 0.19
		1	−20.00 ± 1.81	10.00 ± 0.91

**Table 4:** Frequency change (Δ*f*) and resistance change (Δ*R*) determined in QCM measurement during adsorption of E5 miniprotein at various concentrations.

pH	E5	*c*/g/L	Δ*f*/Hz	Δ*R*/Ω
4.1	NATIVE	0.001	−2.00 ± 0.18	0.00 ± 0.03
		0.01	−5.00 ± 0.45	1.00 ± 0.11
		0.1	−14.00 ± 1.34	2.00 ± 0.18
	AMYLOID	0.001	−4.00 ± 0.42	1.00 ± 0.12
		0.01	−7.00 ± 0.83	2.00 ± 0.21
		0.1	−13.00 ± 1.33	4.00 ± 0.47

**Table 5:** Molecular properties of applied proteins. * [58].

Protein	$M/\frac{g}{\mathrm{mol}}$	Number of AA	Size of Molecule/nm	Number of S-S Bridges	i.e.p./pH
Lysozyme *	14,300	129	4.5 × 3 × 3	4	10.7–11.1
β-lactoglobulin *	18,400	162	3.6 × 7	2	5.4
E5	2783	25	-	-	4.8

## Data Availability

Not applicable.

## Supplementary Materials

The following supporting information can be downloaded at: https://www.mdpi.com/article/10.3390/ijms232113219/s1.
