# Supplementary material for: Comparative Study of the Solid-Liquid Interfacial Adsorption of Proteins in Their Native and Amyloid Forms
Source: Int J Mol Sci. 2022 Oct 30;23(21):13219. doi: 10.3390/ijms232113219 (PMC9656260; doi:10.3390/ijms232113219)
Supplement: Supplementary file 1 [file ijms-23-13219-s001.zip › ijms-1982474-supplementary.pdf]

Supplementary Material

To summarize the results of the ANS fluorescence measurements, the peak areas comparing the native and corresponding amyloid forms at various ANS/protein ratios are shown in Figure S1. There was a notable increase in fluorescence intensity for both proteins, which is higher when higher amounts of ANS are applied, and also increases with protein concentration in the given range. The enhancement ratio was found to be 30–60-fold for lysozyme while about 10-fold for β-lactoglobulin, which clearly indicates the structural changes of proteins providing a hydrophobic environment for the bound ANS molecules following the amyloid formation.

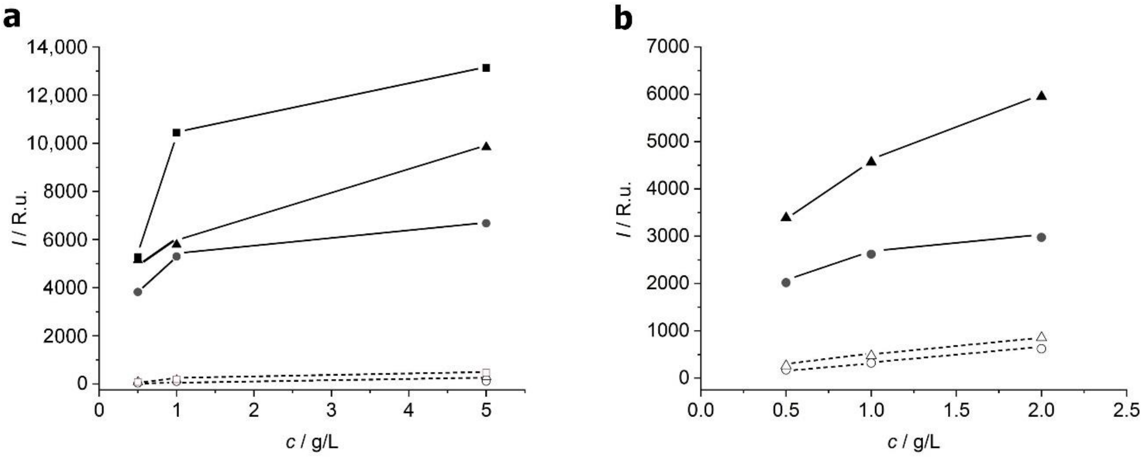

Figure S1. Fluorescence peak area (intensity in water Raman units) of ANS with native (open symbol) and amyloid (filled symbol) lysozyme (**a**) and β-lactoglobulin (**b**) as a function of protein concentration. Concentration of ANS were 2.5 (●), 5 (▲) and 8 mM (■).

Table S1. The ratio of secondary structural elements of the measured proteins in their native and amyloid state was determined from the measured ECD curves using the BeStSel program.

	**Lysozyme**		**β-lactoglobulin**		**E5**	
%	native form	amyloid form	native form	amyloid form	native form	amyloid form
**α-helix**	59	28	43	10	68	0
**antiparallel β-sheet**	0	19	1	28	0	86
**parallel β-sheet**	0	0	19	0	2	0
**turn**	0	0	15	14	0	14
**other**	41	53	22	48	30	0

Table S2. Size of amyloid fibers based on the analysis of the AFM images.

	Lysozyme	β-lactoglobulin	E5
**Fiber thickness / nm**	4.25 ± 0.47	3.46 ± 0.27	3.00 ± 0.25
**Fiber length / μm**	2.20 ± 0.44	3.52 ± 0.39	0.95 ± 0.12

Table S3. Adsorbed mass of proteins at various concentrations and pHs calculated using Equation (3) from frequency changes determined in QCM measurement. Numbers in italic indicate the unreliable (underestimated) mass values due to simultaneous high resistance change (ΔR>3 Ω).

	c / g/L	Δm_A / μg/cm²			
		**pH=2.0**		**pH=7.0**	
		**NATIVE**	**AMYLOID**	**NATIVE**	**AMYLOID**
**Lysozyme**					
	0.001	0.000 ± 0.001	0.000 ± 0.001	0.167 ± 0.018	0.159 ± 0.017
	0.01	0.000 ± 0.001	0.070 ± 0.007	0.218 ± 0.023	0.274 ± 0.022
	0.1	0.035 ± 0.006	0.115 ± 0.012	0.317 ± 0.032	0.347 ± 0.031
	1	0.079 ± 0.009	0.164 ± 0.019	0.493 ± 0.037	0.424 ± 0.036
**β-lactoglobulin**					
	0.001	0.035 ± 0.004	0.018 ± 0.002	0.124 ± 0.013	0.124 ± 0.014
	0.01	0.071 ± 0.009	0.124 ± 0.014	0.299 ± 0.029	0.225 ± 0.023
	0.1	0.159 ± 0.017	0.182 ± 0.019	0.651 ± 0.072	0.299 ± 0.028
	1	0.238 ± 0.021	0.176 ± 0.018	0.353 ± 0.032	0.353 ± 0.032
**E5**		**pH=4.1**			
		**NATIVE**		**AMYLOID**	
	0.001	0.035 ± 0.003		0.070 ± 0.007	
	0.01	0.088 ± 0.008		0.124 ± 0.015	
	0.1	0.246 ± 0.023		0.229 ± 0.023	
